# Editorial: Brain-Computer Interfaces for Perception, Learning, and Motor Control

**DOI:** 10.3389/fnins.2021.758104

**Published:** 2021-10-20

**Authors:** Saugat Bhattacharyya, Amit Konar, Haider Raza, Anwesha Khasnobish

**Affiliations:** ^1^School of Computing, Engineering and Intelligent Systems, Ulster University, Londonderry, United Kingdom; ^2^Department of Computer Science and Electronic Engineering, Artificial Intelligence Laboratory, ETCE Department, Jadavpur University, Kolkata, India; ^3^University of Essex, Colchester, United Kingdom; ^4^TCS Innovations Laboratory, Kolkata, India

**Keywords:** perception, motor imagery, motor learning, brain-computer interfacing, brain activations

This special issue is aimed at offering the latest research outcomes in Brain-Computer Interfaces (BCI) with special reference to perception, learning, and motor control. Perception refers to our ability to sense and interpret the environment in form of a stimuli. Learning is a process to memorize extracts of perceived information and/or their inter-relations, to develop our ability to classify objects from their partial information (attributes) and/or to group (cluster) objects based on a certain measure of their similarity. Motor control refers to our ability to control the motion-related parameters, such as position, velocity, and acceleration of our voluntarily controllable external organs and/or muscles (Pirondini et al., 2017). Although the above terminologies apparently represent different fragments of cognition, they have a precedence relationship from the points of view of their usage. For instance, without perception, learning is impossible. Further, without learning, we cannot perform motor control (Elliott et al., 2011). It may be recalled that children develop their skill of motor control by repeated trials of executing motor actions and their successes and failures.

The motivation of this special issue is to explore the biological underpinnings of perception, learning and motor control from the brain activations captured by electroencephalography (EEG), functional Near-Infrared spectroscopy (f-NIRs), and implantable intra-cortical devices, connected with human/animal brains in the settings of a Brain-Computer Interface (BCI). The special issue includes 16 papers which are summarized below.

Xie, Peng et al. examined the scope of transcranial electrical stimulation on brain activity during motor imagery (MI) activations. The most interesting finding of this research lies in the phenomenon that transcranial current stimulation helps in regulatory brain activity and enhances valid features during non-invasive MI-BCI processing.

Wang et al. proposed a new paradigm for long-term treatment therapy for motor dysfunction caused by neurological injury in the brain. The important aspect of this paper lies in capturing the changes in the brain connectivity caused by short-term neurofeedback. This is perhaps due to the participation of new groups of neurons in the motor learning task with a tendency of rehabilitation by the participating neurons.

Zaer et al. provided a detailed design of an experimental framework for real-time recording and manipulation of neural circuits acquired from intra-cortical electrodes of freely moving animals. The signal acquisition system includes a 64-channel intra-cortical electrode array with a rechargeable battery implanted in the visual cortex to record and manipulate local field potentials. The proposed scheme gives a local signature using a wireless connection to an external network for long-term pre-clinical study of the visual neural circuit after irradiation exposure.

Kim et al. presented an experimental setup to study the effect of subjective emotional changes due to auditory stimuli on the control performance of a P300 based BCI system developed to control an electric light device. Four conditions on the auditory stimuli, such as high valence, low valence, noise, and no sound are used to influence the user to change her emotion, while the user is engaged in controlling the electric light using the P300 based BCI. The paper ultimately arrives at the conclusion that the external influence of emotional stimuli cannot influence the P300 based BCI control. The study thus emphasizes the robustness of P300 based BCI control even when the subject is disturbed by external stimuli.

Rosanne et al. proposed a novel technique of adaptive filtering to improve EEG-based mental workload assessment of ambulant users. The proposed adaptive filter relies on an accelerometer-based referential signal when the subject is engaged in multi-attribute decision-making tasks, while walking/jogging on a treadmill. In presence of the proposed adaptive filter algorithm, the authors obtained high classification accuracy of 95% using a random forest-based 2-class mental workload classification, when experimented on ambulant Users.

Li et al. proposed a self-organized graph neural network (SOGNN) for cross-subject EEG-based emotion recognition. The novelty in the proposed work lies in the dynamic construction of the graph neural network by a self-organized module. The performance analysis is undertaken by considering variations in the graph construction techniques. Visualization of the graph structure learned by the proposed model coincided with the previous neuroscience research. This implicates the effectiveness of the proposed model in the context of neuroscience.

Lau et al. examined the neuroplasticity changes in stroke survivors due to the training of hand movements by a BCI-guided robot arm. The study includes neural modulation in functional connectivity and the clinical improvements immediately after and 6 months after the training of the subjects by the assistive BCI system. The experimental results indicate that neural activity in sensory-motor and frontoparietal regions, which are highly involved in BCI-guided training, show significant changes in functional connectivity.

Derzsi examined the scope of spectral power density and phase coherency features to detect Steady-State Visual Evoked Potential (SSVEP) signals. It appears from the experiments that phase coherency features are most sensitive in the detection of weak signals such as SSVEP.

Khan and Hasan studied bimodal fusion of electroencephalography (EEG) and functional Near-Infrared spectroscopy (f-NIRs) signals to improve performance in motor-task classification. Here, the authors proposed Multi-resolution Singular Value Decomposition (MSVD) to achieve system- and feature-based fusion. Finally, the authors employed tree and k-nearest neighbors (k-NN) algorithm-based classification to determine the efficacy of the bimodal fusion.

The tangent Space Mapping (TSM) algorithm is a well-known technique to recognize multi-class motor imagery (MI). However, the EEG features induced by MI mental activities of the subjects being different, selection of subject-specific discriminative EEG frequency components has an important role in the recognition of multi-class MI. Wu et al. extended the classical TSM algorithm by incorporating multi-scale filter banks to recognize the tangent space features in each sub-band. Finally, a Linear Support Vector Machine (LSVM) classifier is used to classify the MI. The authors claim that the classification accuracy of the extended algorithm is increased by 3.36% with respect to the traditional TSM algorithm for MI classification.

Xie, Cao et al. made an interesting and unusual claim that an introduction of moderate auditory noise enhances the BCI performance in the visual modality. This is referred to as cross-modal stochastic resonance (SR) theory. Although cross-modal SR theory has been tested in different sensory systems, its application in BCI is novel. Here, the authors employed Fast Fourier Transform (FFT) and Canonical Correlation Analysis (CCA) to evaluate the influence of noise in the periodic components of the visual response. Directed Transfer Function (DTF) was used to investigate the functional connectivity patterns, and the flow-gain value is used to measure the degree of activation of the specific brain regions in the information transmission process. The flow-gain maps demonstrated that moderate-intensity in audio noise activates the brain area to a great extent. Further analysis by weighted phase-lag index confirms that phase synchronization between visual and auditory regions in presence of auditory noise is significantly enhanced.

Simar et al. deals with an interesting problem on the detection of festive or violent intent of subjects before execution of their actions in interpersonal interactions. The authors here develop a classifier based on covariance matrix and Riemannian geometry that can effectively discriminate neutral, festive, and violent mental states on the basis of non-invasive EEG signals in both the actor and the observer participants. This research outcome may serve as an important component for the next generation of social interaction among people with portable EEG devices on their heads. The observers in such interactions may get enough time to keep themselves prepared to move away before the actual execution of the violent action by the actor.

Liu et al. proposed a novel approach to design motor imagery classifiers using Convolution Neural Network (CNN) with parallel spatial and temporal self-attention modules. The spatial self-attention module is designed to capture the spatial dependencies between channels of motor imagery EEG signals. It updates the features of each channel by considering the weighted sum of the same feature of other channels. Such updating keeps away the possibility of individual channel features being severely affected by artifacts. The temporal self-attention, on the other hand, is employed to encode the global temporal information into features over each sampling time-steps, to obtain high-level temporal features of the MI EEG signals in the time-domain. The proposed CNN model is tested in position control of drones using MI EEG signals of the experimental subject.

Roy et al. proposed an interesting solution to the long-standing inter-subject transfer learning problem in the MI-based BCI. The primary bottleneck in transfer learning under BCI settings is due to high inter-subject variability in brain signals related to MI. Here, a Convolution Neural Net (CNN) based deep learning is proposed with provisions for inter-subject continuous decoding of MI-related EEG signals using the novel concept of Mega Blocks for adapting the network against inter-subjects' variability. The parameters of the Mega Blocks are optimized using Bayesian hyper-parameter optimization. The proposed CNN-based architecture would serve as an important module in the development of the calibration-free next-generation classifiers with the flexibility of inter-subject continuous decoding of motor imageries.

Traditional MI-based BCI systems generally employ left hand, right hand, and foot motor imageries as 3 basic commands in the BCI design. However, to develop bigger BCI systems, we may consider simultaneous activation of 2 or more basic commands, and thus generate 8 possible commands including the rest condition as well into account. The main limitation of the multi-command BCI systems is the difficulty in decoding the commands due to constraints to maintain adequate spacing among the corresponding sources, and also due to stochastic noise of the signal sources. Lindig-Leon et al. proposed a solution to the above problem by transforming the 8-class problem into a set of 3 binary problems to facilitate the use of proposed multi-label Common Spatial Patterns (CSP) algorithms. Two different realizations of multi-label CSP algorithms, called MC2CMI and MC2SMI are proposed in the paper. Both the algorithms return 3 sets of features, one for the left hand, one for the right hand, and the rest for foot MI. Finally, the 3 sets of features are merged together into a vector to predict the user intention by employing an 8-class Linear Discriminant Analysis (LDA) classifier.

Fathima and Kore in this special issue has dealt with another interesting problem on feature selection and channel selection in EEG-BCI systems using optimization algorithms. Here, the authors demonstrate the formulation of a single objective, multi-objective, and constrained optimization objective function for different types of BCI applications, including control problem in prosthetic arms, gaming, and many others. The importance of the paper lies in the thorough review of existing works along with authors' own contributions in the choice of suitable objective functions for a given problem.

The editors strongly believe that this issue will be useful to the BCI researchers, doctoral students, and BCI-developers, who are curious to employ MI BCIs in different applications. The editors are grateful to the authors for submitting their valuable contributions to this special issue, and also to the publisher to allow them to edit this interesting volume.

## Author Contributions

SB, AKo, HR, and AKh contributed in managing the review process and in drafting the editorial. All authors contributed to the article and approved the submitted version.

## Conflict of Interest

The authors declare that the research was conducted in the absence of any commercial or financial relationships that could be construed as a potential conflict of interest.

## Publisher's Note

All claims expressed in this article are solely those of the authors and do not necessarily represent those of their affiliated organizations, or those of the publisher, the editors and the reviewers. Any product that may be evaluated in this article, or claim that may be made by its manufacturer, is not guaranteed or endorsed by the publisher.
